# Steps towards COVID-19 suppression

**DOI:** 10.1186/s41232-020-00120-z

**Published:** 2020-06-22

**Authors:** Hideyuki Okano, Ken-ichiro Seino

**Affiliations:** 1 Department of Physiology, Keio University School of Medicine, Tokyo, 160-8582 Japan; 2 Division of Immunobiology, Institute for Genetic Medicine, Hokkaido University, Sapporo, Japan

From a historical perspective, pandemics have been repeatedly caused by different pathogens, such as plague in the fourteenth century and cholera in the nineteenth century. Since the twentieth century, we have had the Spanish flu almost 100 years ago, the worst infectious disease in human history, and now the COVID-19 which is caused by SARS-CoV-2 infection [1–3]. COVID-19 pandemic is going to profoundly change the health, medicine, economy, and daily life of humanity around the world in this century. It is obvious that the suppression of COVID-19 is an urgent task for all humankind, but even if this is achieved, it has been pointed out that new pandemics may be triggered more frequently in the future due to not only outbreaks of new zoonosis caused by ecological, behavioral, or socioeconomic changes [4] but also the spread of various pathogens that have been isolated in the past as the permafrost in Siberia thaws due to global warming. Based on these scientific evidences and the latest predictive technologies, there is a need to establish diagnostic, therapeutic, and prophylactic methods, including vaccines, for COVID-19.

In this theoretical series reviews, although it is not possible to cover all of the subjects mentioned above, we entitled this “COVID-19: its pathogenetic mechanisms and potential therapeutics” and invited leading researchers on COVID-19-related studies from the perspective of inflammation research and regenerative medicine and translational research, which are this journals’ specialties, in addition to the basic properties and epidemiology of SARS-CoV-2 as follows:

The basic properties and epidemiology of SARS-CoV-2:

*➢ Dr. Nureki* and colleagues will review the basic RNA and structural biology of SARS-CoV-2.

*➢ Dr. Nakagawa* and colleagues will review the comparative virology and genetics of SARS-CoV-2.

*➢ Dr. Nakatani* and colleagues review epidemiology and public health of COVID-19.

Pathological mechanisms of COVID-19: involvement of inflammation

*➢ Dr. Seino* and colleagues review macrophage activation syndrome (MAS).

*➢ Dr. Murakami* and colleagues review IL6-mediated cytokine storms associated with COVID-19.

Diagnosis of COVID-19 and detection of SARS-CoV-2

*➢ Dr. Kanuka* and colleagues review their series of original studies regarding on intensive diagnostic management of coronavirus disease 2019 (COVID-19) in academic setting and also discuss their challenge and future.

Potential therapeutics for COVID-19

*➢ Dr. Terai* and colleagues reviewed on therapeutic potential of mesenchymal stem cells (MSCs) and their exosomes in severe novel coronavirus disease 2019 (COVID-19) cases [5]. They described about basic aspects and mechanisms of action of MSCs and exosomes and potential of MSCs and exosome therapy in severe cases of COVID-19 in recently initiated or planned clinical trials registered in eight countries on ClinicalTrials.gov.

*➢ Dr. Hashizume* describes their current investigation on clinical trials for COVID-19-associated cytokine storm with tocilizumab.

Prophylactic methods for COVID-19

*➢ Dr. Miyazawa* and colleagues describe about herd immunity for COVID-19 pandemic.

In addition to these invited reviews, we have opened a call for submissions reporting ongoing medical research in wide range of topics related to COVID-19, in the form of original articles, review articles, or opinion papers on the following topics (https://www.biomedcentral.com/collections/COVID19IPMPT).

• Molecular biology and virology of SARS-CoV-2

• Development of new diagnosis of COVID-19 infection

• Cytotoxicity and pathogenetic mechanisms of COVID-19

• COVID-19-related inflammatory response including cytokine storm

• Drug development for COVID-19-related disorders using stem cells and organoid cultures

• Development of new interventions for COVID-19-related disorders

• Development of vaccines for COVID-19

• Clinical trials for COVID-19-related disorders

Here, we would like to express sincere gratitude to the distinguished researchers who contributed to this theoretical series reviews and sincerely hope that these review articles will provide novel insights for the suppression of COVID-19 as well as to the researchers in the broad field of inflammation and regeneration.
